# Self-Determination in Health Research: An Alaska Native Example of Tribal Ownership and Research Regulation

**DOI:** 10.3390/ijerph14111324

**Published:** 2017-10-31

**Authors:** Vanessa Y. Hiratsuka, Julie A. Beans, Renee F. Robinson, Jennifer L. Shaw, Ileen Sylvester, Denise A. Dillard

**Affiliations:** 1 Southcentral Foundation Research Department, 4085 Tudor Centre Dr., Anchorage, AK 99508, USA; vhiratsuka@scf.cc (V.Y.H.); rrobinson@scf.cc (R.F.R.); jshaw@scf.cc (J.L.S.); dadillard@scf.cc (D.A.D.); 2 Southcentral Foundation Executive and Tribal Services, 4501 Diplomacy Drive, Anchorage, AK 99508, USA; isylvester@scf.cc

**Keywords:** community review, Alaska Native, tribal, ethics, Native American, research, research conduct, trust, accountability

## Abstract

Alaska Native (AN) and American Indian (AI) people are underrepresented in health research, yet many decline to participate in studies due to past researcher misconduct. Southcentral Foundation (SCF), an Alaska Native-owned and operated health care organization, is transforming the relationship between researchers and the tribal community by making trust and accountability required features of health research in AN/AI communities. In 1998, SCF assumed ownership from the federal government of health services for AN/AI people in south central Alaska and transformed the health system into a relationship-based model of care. This change reimagines how researchers interact with tribal communities and established community oversight of all health research conducted with AN/AI people in the region. We describe the SCF research review process, which requires tribal approval of the research concept, full proposal, and dissemination products, as well as local institutional review board approval, and a researcher-signed contract. This review evaluates research through the lens of tribal principles, practices, and priorities. The SCF example provides a framework for other tribes and organizations seeking to reshape the future of health research in AN/AI communities.

## 1. Introduction

Self-determination is the process and authority by which nations establish allegiances and pursue independent social, economic, and cultural aims. The Indian Self-Determination and Education Assistance Act of 1975 (P.L. 93-638) codified self-determination of Alaska Native and American Indian (AN/AI) people by allowing tribes and tribal organizations to contract directly with the United States federal government for health and education grants and thereby exercise greater control over their own welfare [1,2,3,4]. Many tribes and tribal organizations have since exercised the right to own and manage federally funded health services and programs under this process of self-governance [5,6,7,8,9]. Awareness of health disparities experienced by the AN/AI community compared with other U.S. populations has also spurred many tribes and tribal organizations to invest and engage in health research [7,10,11,12].

AN/AI people across the lifespan consistently rank lowest in the U.S. in a wide range of health outcomes, as well as many social determinates of health [13,14,15,16,17]. A long history of government policies against tribes in the U.S.—including military aggression; deliberate introduction of alcohol and disease; religious schools, federal- and state-run boarding schools; and displacement, internment, and forced relocation—has had lasting negative impacts on the physical, mental, and community health of the AN/AI population for multiple generations and caused intergenerational trauma that contributes to substance misuse and interpersonal violence in AN/AI communities today [10,18]. Concurrently, unethical health research as well as the continued misalignment of perceived benefits and risks of health research between health researchers and AN/AI communities have led tribal leaders to recognize a need for methods of tribal oversight to guide and regulate health research conduct in tribal communities [2,19].

The Indian Health Service (IHS), the federal agency within the United States Department of Health and Human Services responsible for providing medical and public health services to members of federally-recognized AN/AI tribes [3], has played an important role historically in oversight and provision of AN/AI health services and research [20,21]. The IHS established an Institutional Review Board (IRB) for each of the 12 regional administrative offices and a national level IHS IRB. As set forth under the Code of Federal Regulations, Title 45, Part 46, which is entitled Protection of Human Subjects (hereafter referred to as 45 CFR 46), IRBs regulate all research involving human subjects. IRBs follow general ethical principles that are applied to research with human subjects and exist to protect the autonomy, safety, privacy, and welfare of individual human research subjects. Federal code requires research that occurs within an IHS facility, with IHS staff, or uses IHS resources is reviewed by the area IHS IRB and the National IHS IRB, across IHS areas. 

As tribes have assumed ownership of health services under Public Law 93-638, some tribes continue to rely on the area IHS IRBs, but an increasing number of tribes have formed tribal IRBs to provide this oversight [22]. Tribal IRBs typically support local ethical principles, norms, and cultural protocols, preserve sovereign rights, and protect tribal citizens from research harms at both individual and group levels. In addition to protecting individual rights of research participants, tribes exercise sovereignty to govern the impact of research on groups and communities, such as AN/AI research participants’ families, the tribe as a whole, and, to some degree, indigenous people nationally and internationally [23]. Such group level impacts of research are not integral to general ethical principles, such as the Belmont Report that governs IRB review, yet are critical aspects of the perceived harms and benefits of research in AN/AI communities [24]. Recent changes in the Common Rule, along with the creation of a new tribal Health research Office at the National Institutes of Health (NIH) provide additional recognition and support to AN/AI communities in developing and conducting local community-based governance and oversight of research conducted with tribal peoples and on tribal lands/facilities [25].

Tribes and the tribal health organizations they manage have exercised various approaches to overseeing research conducted with tribal members and their communities [7,11,18,20,22,23,26,27,28,29,30,31,32]. Some AN/AI communities that have elected to self-govern health care have enacted Tribal IRBs or other policies to oversee health research, whereas others have implemented tribal research review bodies to work alongside the IHS Area IRB [12,29]. The latter option, of establishing an independent review process focused on local tribal values and conducted by individuals granted authority under AN/AI tribal governance, is an example of self-determination in tribal healthcare [7,12,20,33].

In this paper, we describe the research review process developed by an AN-owned and operated tribal health organization to regulate research within and for a culturally-diverse and geographically-expansive AN/AI community. This case example is provided not only for other AN/AI entities who are considering exercising sovereignty over health research, but also for the research community as a whole to better understand the overall intent and nature of tribal review.

## 2. Southcentral Foundation: Self-Determination in Alaska Native Health

Southcentral Foundation (SCF), a tribally owned and operated health care organization in Anchorage, Alaska, was incorporated in 1982 under the tribal authority of the Cook Inlet Region, Inc. (CIRI) [8]. SCF service delivery area includes Anchorage, the Matanuska-Susitna Valley, and 55 rural Anchorage service unit villages [34]. The first compact agreements under Public Law 93-638 between SCF and the IHS included dentistry, optometry, and community health and injury control services [8]. The provision of other health care services remained with the IHS. After decades of experiencing limited access to primary care services, long waits for care, lack of preventative services and behavioral services, and high turnover of clinical staff, SCF took steps to assume ownership and management of the entire primary care system from the IHS and, in 1998, initiated an AN/AI driven system-wide redesign [35]. The SCF corporate mission and vision were developed to reflect the health and wellness needs and the preferences of the tribal communities SCF serves. The SCF corporate mission is “working together with the Native Community to achieve wellness through health and related services” and the SCF corporate vision is “a Native Community that enjoys physical, mental, emotional and spiritual wellness” [34]. To further emphasize the philosophical change from a federally-operated facility to a tribally-operated facility, the term “patient” was replaced with “customer-owner” to signify that Alaska Native people receive care as customers from a healthcare system they also own [8].

In addition to transforming the delivery of health services, the shift from federal to tribal management presented an opportunity to change the way health research occurs and to develop policies for oversight of research occurring under the tribal authority extended to SCF. Guided by SCF’s goals of shared responsibility, commitment to quality, family wellness, and operational excellence, the SCF Board of Directors, executive staff, researchers, and legal counsel co-developed a series of corporate policies and procedures to govern all aspects of health research (Table 1). Like healthcare service delivery, the planning and conduct of health research at SCF is grounded in self-determination.

First, a document outlining guidelines for researchers (Table 2) was created. The guidelines for researchers contain four areas of note: (1) alignment to SCF organizational tenets; (2) quality of research design; (3) depiction and involvement of Alaska Native people; and (4) topical areas considered sensitive with the tribal organization. Within the alignment section of the guidelines, researchers are directed to describe their research approach using the lens of SCF’s mission, vision, health priorities, and values. SCF leadership directly states in the Guidelines for Researchers document that the organization works to ensure that the community members are not overly researched as a population without commensurate benefit and view the time and energy devoted to research as a valuable and limited resource. Within the quality of research design section of the guidelines, researchers are directed to balance the collection of both strengths/protective factors and risk/pathology variables as well as transparently describe data collection, analysis plans, and dissemination with attention to how AN/AI people will learn about results. Within the depiction and involvement of Alaska Native people section of the guidelines, researchers are provided preferred language to use in their research projects and dissemination efforts and given questions to consider to avoid outright racial stereotyping of AN/AI people, unintentional paternalistic descriptions of AN/AI people or communities, and tokenism in inclusion of AN/AI people in the research process. Finally, there is a section in the guidelines for researchers listing areas SCF leadership has determined to qualify as sensitive research areas and thus deserving of additional scrutiny.

SCF leadership also communicates community established health priorities with researchers to encourage research that aligns with the effort to reduce health inequalities of significance to the AN/AI community. Beyond ensuring that research in the tribal health system occurred in culturally ethical and acceptable ways, the review process had two primary goals: (1) to inform clinical practice in a timely manner by ensuring that findings are communicated to relevant staff; and (2) to contribute to the canon of knowledge in the health sciences. Research findings are able to be translated into public health and clinical changes to impact healthcare delivery and health outcomes upon verification of improved efficacy and community acceptability.

## 3. Establishment of the Southcentral Foundation Research Policy Procedure

In 2005, SCF developed a research policy to provide direction for all research activity impacting SCF customer-owners, occurring within SCF facilities, and/or conducted by SCF employees. This policy and the previously mentioned guidelines for researchers express SCF’s intent to support research that is in the best health/wellness interest of customer-owners while acting to protect the safety and well-being of the community. SCF seeks to protect and preserve AN/AI cultures and to ensure research activities are conducted in a way that is respectful and does not harm AN/AI people or AN/AI culture. The process recognizes that the community involved in research can be defined as the AN/AI community actively participating in research as well as the AN/AI community potentially impacted by the conduct and/or findings from that research. The SCF Research Policy and Procedure equally prioritizes both the clinical and public health relevance of research with cultural and community relevance. In other words, an inquiry must be considered relevant and worthwhile in the eyes of *both* the cultural/community *and* the scientific stakeholders to be approved. Additionally, as directed by this policy, research conducted within the clinic setting must not impede the provision of health and wellness services. This requirement necessitates that researchers engage operational and clinical leaders and other key stakeholders early in project design, unlike much previous health research which often progressed to data collection before any contact was made with clinic staff or leaders. This early stakeholder engagement not only makes certain that the research does not interfere with clinic operations, but aides in the subsequent dissemination and implementation of research findings by identifying and ameliorating potential barriers long before they can become a problem. The early engagement also helps to co-create research questions with maximum local applicability, leading to enhanced sustainability of project findings through establishment of trustworthy relationships at project onset.

In 2006, SCF created a research department to assist in the implementation of the research policy and guidelines in the community and to conduct health research on behalf of the AN/AI community from *within* the health system. The SCF Research Department addresses a wide variety of medical and behavioral health topics aligned with the corporate family wellness objectives, thereby ensuring that the research benefits SCF customer-owners by addressing community-identified health priorities. The SCF Research Department consists of 25 interdisciplinary doctoral, master’s-, bachelor’s-, and pre-baccalaureate-level staff members, 76% of whom are of AN/AI descent. Doctoral-level staff, which includes individuals with training in psychology, medical anthropology, pharmaceutics, and public health, assist tribal and clinical leadership with research review. SCF leadership strategically planned for the SCF Research Department staff to work in partnership with external groups (e.g., university-based researchers) to support the latter’s respectful and sustained engagement with tribal communities. Through collaboration with SCF Research Department staff, external researchers would be exposed to the practice of indigenous methodologies (e.g., oral story, locating of self, local relevancy, etc.) while SCF staff concurrently gain exposure to specialists in particular health research areas allowing for exchange of knowledge while working towards research in advancement of AN/AI health.

All health research activities (recruitment, querying of medical record data, data collection, etc.) occurring within the SCF service delivery area must have approval from SCF prior to study initiation. The Alaska Area IRB (AAIRB) of the IHS acts as the primary IRB for all research proposals to determine if individual human subjects are protected under federal laws and guidelines. In addition, the AAIRB adds a stipulation to approval requiring all proposals involving SCF customer-owners be submitted to and approved by the SCF tribal review process. The volume of research activities under review is substantial; 138 submissions were reviewed in the SCF tribal review process from January 2016 through December 2016.

## 4. The SCF Review Process

**Figure 1:**
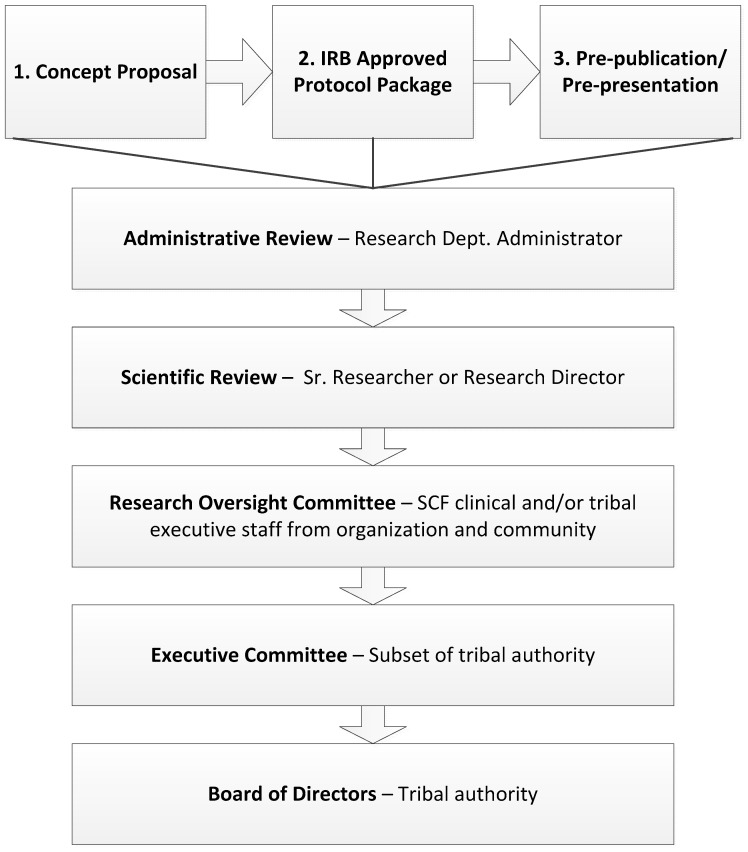
Southcentral Foundation Tribal Review Process.

The SCF tribal research review process involves a multi-level process of administrative, scientific, and tribal review. First, each submission receives an administrative review and a scientific review prior to being reviewed by three groups of clinical and tribal leadership, including the: Research Oversight Committee (comprised of twelve SCF health system leaders), Executive Committee (comprised of four members of the Board of Directors), and Board of Directors (comprised of seven CIRI shareholders) (Figure 1). The preliminary (administrative and scientific) reviews verify the submission is (1) complete, and (2) includes the elements outlined in the Guidelines for Researchers, specifically that the investigator has used person-first language, already engaged appropriate clinic and program staff, and approached the research in a scientifically sound and culturally respectful manner. Each subsequent level of review is comprised of SCF clinical and/or tribal executive staff representing viewpoints from across the tribal health organization and community. Reviewers ensure that tribal authorities and customer-owner rights are respected by considering the following issues and questions delineated in the SCF Guidelines for Researchers:

- Alignment with SCF values and health priorities;
- Potential benefits and harms to SCF customer-owners and AN/AI people including potential financial benefit and use of SCF resources;
- Quality of research design including how participants and AN/AI people will learn about the results;
- Depiction and involvement of AN/AI people including whether the approach or findings could potentially stigmatize the AN/AI community or the AN/AI health system as well as involvement of AN/AI people in the design and/or conduct of the research, including potential authorship or co-authorship of publications;
- Researchers expected to use “person/people first” language (e.g., “individuals with chronic mental disorders” rather than “the chronic mentally ill”);
- Impact on systems and service delivery;
- Type of information that will be sought from individuals or other participation involving individuals, including the donation of samples; and
- Type of information concerning the culture, religion and customs and practices of AN/AI people, either historical or contemporary.

Each step of the review process is designed to allow SCF customer-owners, clinical providers, health system and tribal leaders, and administrative staff to work as partners with researchers in developing a balance between expected immediate and long-term benefits of the proposed research to participants and AN/AI people and risks associated with the research. Some risks considered include the physical or psychological well-being of individual participants and possible adverse impact on the cultural, social, economic or political well-being of the AN/AI community. Reviewers consider SCF resources needed for the research and the impact of conduct of research on SCF health care delivery and space, responsibilities of SCF staff, and costs to be borne by SCF (e.g., electronic health record query and dataset creation using analyst time that would normally be used to generate clinical reports for clinical quality assurance and improvement). Researchers are also expected to provide the organization with a plan for dissemination of findings that demonstrates study participants and AN/AI people are engaged and informed throughout the conduct of the research project and after results are complete. SCF encourages and prioritizes AN/AI representation in all aspects of research projects in the region, preferably by a member of our own staff to certify research is conducted respectfully. The AN/AI employee will be included in the writing group for abstracts and manuscripts submitted for publication and will be responsible for assisting in SCF use of research results within clinical practice when applicable.

The SCF research review process includes: concept proposal, proposal review by the AAIRB, proposal review by SCF, continuing annual review by the AAIRB and SCF, and pre-publication review by SCF (Figure 1). The SCF concept proposal is the first step in the research review process. In this phase, the researcher submits a single page narrative outlining the scope of the proposed project, their aims and hypotheses, the risks and benefits of the study, and their proposed study team. SCF reviewers use the concept proposal as an opportunity to determine if the proposed project fits within the community identified health priorities. If there is a discrepancy between organizational goals and researcher goals, the SCF Research Department staff opens a dialogue with researchers to attempt to better align the research concept with the needs of the tribal community. If the Principal Investigator is unwilling to adjust the scope of the project to meet the contingencies placed by SCF leadership, the research project will not be approved and it will not occur.

Proposal review is the next step in the review process, and this occurs after a Principal Investigator has obtained concept approval and AAIRB approval for the proposed research. In this phase, the Principal Investigator submits their research protocol and all associated documents (e.g., recruitment materials, informed consent forms, data collection instruments, electronic health record data query requests, data analysis plans, key personnel biosketches, etc.) for consideration by the community level reviewers. SCF reviewers use the proposal review as an opportunity to examine the research approach to recruitment, enrollment, data collection, data retention, analysis, dissemination to participants and peer-review, and intended use of SCF resources. If researchers outside of the SCF health system are conducting health research, reviewers facilitate communication between the researchers and the community in which the research is being conducted to assist in village level approval if needed. If there is a discrepancy between any aspect of the proposed research project and community ethics and practices, the SCF Research Department staff opens a dialogue with researchers to better align the proposal with the needs of the tribal community. If the Principal Investigator is unwilling to adjust the research to meet the contingencies placed by the SCF Board of Directors, the research project will not be approved and research will not occur at SCF.

Following proposal approval, the Principal Investigator is provided a researcher agreement, a set of standard stipulations to be followed by researchers whose proposals have been approved [21]. The research agreement was developed as a contract between the Principal Investigator and SCF to document that the researcher and their research team agrees to:

- abide by SCF codes of conduct and ethics,
- promptly provide notice of any significant changes to the research plan and obtain SCF’s approval of such changes,
- maintain confidentiality of data as appropriately applied to individuals and, where necessary, to families, communities, and SCF itself for the life of the project, and
- assure that all data collected during the research project including biological material are the property of SCF and will be returned to SCF when the research is complete, unless otherwise agreed to with the researcher and that any financial benefit or ownership of product developed will be the property of SCF unless otherwise agreed to with the researcher.

It is important to note that the agreement can be modified if all parties come to a consensus. For instance, the researcher agreement may need to be altered to assure that data and biological material are the property of the village in which the data were collected if that is the interest of the tribal leadership of the village. The modification would then be reviewed by SCF legal counsel prior to agency finalization. Individuals who are unwilling to commit to the SCF researcher agreement, are not allowed to conduct their proposed research with and for tribal communities SCF serves.

Pre-publication review of manuscripts and abstracts is the final aspect of SCF review of research. In this phase, per the SCF researcher agreement, any abstract describing results of this research must be submitted for approval by SCF tribal review before submission for presentation at a conference. All manuscripts describing results of research conducted must be submitted for final approval by SCF Board of Directors prior to submission for publication. The SCF reviewers use the pre-publication review step to provide protection of AN/AI people in the dissemination phase of the research project, a particular concern as conference abstracts and peer-review manuscripts are available to the public in perpetuity. Pre-publication review allows for an AN/AI perspective to be included to foster a non-stigmatizing and accurate description of the population and setting.

## 5. Case Study—Genetics and Tobacco Cessation Treatment in an Alaska Native Community

The SCF Research Department operates a Native American Research Center for Health that includes a research project “Genetics and Tobacco Cessation Treatment in an Alaska Native Community”. The research project was developed to provide AN/AI communities with sufficient information about pharmacogenetics to determine its utility and potential value to guide tobacco cessation treatment, identify potential genetic variations that could impact treatment, and determine potential utility of these tests to guide tobacco cessation treatment in the AN/AI community. The Principal Investigator (Renee F. Robinson) submitted a concept proposal on the project in January 2012, which was approved by the SCF Board of Directors in March 2012, a two-month process from submission to concept proposal approval. A protocol for the project was submitted to the AAIRB in December 2012 and received approval in April 2013, a four-month process from AAIRB submission to approval. Immediately following AAIRB approval, a proposal for the project was submitted to SCF and the proposal was approved by the Executive Committee of the SCF Board of Directors in June 2013, a two-month process from proposal submission to approval. Immediately following approval of the proposal, the Principal Investigator signed a Researcher Agreement. The research team of the project had preliminary project findings and requested review of an abstract for presentation on December 2013 and received approval in February 2013, a two-month process. On average, the SCF review process takes two months from submission to approval.

## 6. Conclusions

This paper provides an example of the health research review process at Southcentral Foundation, one of 32 tribal health organizations in the state of Alaska. This review process was developed over decades of experience with researchers both within the tribal health system, from University systems and other institutions. The health research review process operated by tribal health organizations in Alaska involves coordination across multiple sovereign nations and communication of the health priorities of the communities to allow for research that is community relevant in addition to being scientifically significant.

Tribes and tribal organizations have the opportunity to take the research review process into their own hands to provide enhanced protections to the tribal communities they serve. This review process seeks to support tribal people beyond those directly under the tribal authority of SCF as results from one AN/AI community’s participation in health research are often generalized to all AN/AI people. Although tribal IRBs are an excellent avenue to exercise a tribe’s sovereign right to oversee research taking place in the tribal community, tribal IRBs are usually required to follow federal regulations as well as tribal policy developed to address review of research. A community-level research review committee offers an additional level of oversight that leaves the federal regulatory responsibility of human subject protections with a traditional IRB, and allows for an in-depth review of research following policy developed at the tribal level.

## Figures and Tables

**Table 1:** Incorporation of Southcentral Foundation Key Points in the research approval process.

Key Points	Shared Responsibility	Commitment to Quality	Family Wellness
Concept Proposal	The research approach respects the diverse and unique cultures and histories of Alaska Native people. Research implications have direct benefit to Alaska Native people.	The research approach considers current and planned health services and staff within the Alaska Native community. Where possible Alaska Native staff will be trained and actively involved.	Research outcomes are in alignment with health objectives, mission and vision of SCF. Research has direct clinical or community implications for prevention or improvement of the mental, spiritual, or physical health.
Proposal	Research uses engagement methods to enhance trusting relationships between the researcher and the Alaska Native community. SCF works to ensure customer-owners are not overly researched as a population without commensurate benefit.	A plan is in place for disseminating findings to Alaska Native people. The research plan is respectful of Alaska Native individuals and communities.	Research purpose is to improve health and systems of care for Alaska Native people. Research approach and instrumentation emphasizes wellness and multiple dimensions of health.
Researcher Agreement	Data and specimens collected during the research project are the property of SCF and will be returned to SCF. Data sharing, specimen sharing, and changes in research approach are subject to SCF prior approval.	The researcher and their team will abide by SCF policies. The researcher and their team will maintain individual, family and community confidentiality for the duration of the project.	Researchers working on projects involving youth will undergo background checks.
Pre-publication/Presentation Review	When possible, Alaska Native people will co-present study findings and serve as manuscript authors.	Findings are provided directly to health system and clinical and tribal leadership.	Findings utilize the SCF Guidelines for Researchers.

**Table 2:** Southcentral Foundation Guidelines for Researchers.

**Alignment with Southcentral Foundation Vision, Mission, Key Points, Goals, and Objectives**	
Reference Southcentral Foundation’s key points, goals, and Family Wellness Corporate Objectives. The following examples depict research approaches with strong alignment: Research on problems and issues of special interest to the Alaska Native community, namely the Family Wellness Corporate Objectives. Research designed to evaluate the efficacy and effectiveness of current health practices and processes. Research with the intended use of improving health and systems of care. Research using a community-based participatory research approach or methods. Research emphasizing wellness and multiple dimensions of health. In addition, the potential benefits and harms are carefully considered as well as the overall importance of the project. Southcentral Foundation works to ensure customer-owners are not overly researched as a population without commensurate benefit and views the time and energy devoted to research as a valuable and limited resource. Is this important? Do the results matter? (So what?) To whom? Are risks to Alaska Native people considered and sufficiently addressed? How will Alaska Native people benefit? Are the benefits accurately described? Are Alaska Native people being used as test subjects without visible benefit? What are you (investigators) planning to do with the information? (Propose new treatments? New diagnostic practices?) Is there likely to be financial benefit from this research? To whom? How are Southcentral Foundation employees and programs which may be impacted by the research involved in the design? Is the effort a good use of Southcentral Foundation and Alaska Native Medical Center resources?	
**Quality of Research Design**	
Is the design of the study clearly described? Are all the important variables included? Are strengths/protective factors considered in addition to pathology/risks? Are there valid comparisons or controls where appropriate? Are there enough people in the study to show significant results? Do the authors limit their conclusions to the group studied? Are limitations acknowledged and described? Have statistical tests been used when appropriate? Are there enough data and are the data clearly represented? Are consent forms and recruitment materials clearly written? What is the source of funding? How will results be applied and shared? How will Alaska Native/American Indian people learn about the results?	
**Depiction and Involvement of Alaska Native People**	
Are Alaska Native people depicted in an inaccurate or stigmatizing light? Are there racial stereotyping or generalizations? Are Alaska Native people described in a paternalistic manner? Is the language regarding tribal affiliations appropriate? How are Alaska Native people involved in the design of the study? Are the contributions of Alaska Native researchers recognized? Does the investigative team include individuals who have worked with Alaska Native/American Indian people? Here are some specific examples of preferred language uses:	
We like to see:	Rather than:
Alaska Native people	Natives *
Bacterium or virus	germs
Alutiiq, Haida, Tsimshian, Tlingit, Yup’ik, Inupiaq, Athabascan, Eyak, Unangax	Eskimo, Indian, Aleut
**Topical Areas Considered Sensitive**	
Descriptions of alcohol and substance abuse, domestic violence, suicide, sexual behavior Specific cultural issues including death and dying, treatment of elders, and historical customs Research involving youth Researchers must undergo background checks and finger printing Southcentral Foundation Board of Directors will be sensitive to anything involving child abuse or sexual behaviors Parental consent and youth assent will be obtained.	

* Additional terms, including customer-owner, patient, and AN community should be used in circumstances in which they are appropriate. Although AN people is preferred over AN/AI people when referring to studies that take place in the state of Alaska, the use of AN/AI people is appropriate when referring to studies that take place both in Alaska and other states.
